# Stress Corrosion Cracking of Ti-6Al-4V ELI Titanium Alloy in 3.5 wt.% NaCl Solution

**DOI:** 10.3390/ma19173572

**Published:** 2026-08-23

**Authors:** Qing Zhao, Aifeng Zhang, Zhengquan Wan, Yafei Wang, Chengqi Sun

**Affiliations:** 1 China Ship Scientific Research Center, Wuxi 214082, China; 2 Taihu Laboratory of Deepsea Technological Science, Wuxi 214082, China; 3 Institute of Mechanics, Chinese Academy of Sciences, Beijing 100190, China; 4 School of Engineering Science, University of Chinese Academy of Sciences; Beijing 100049, China

**Keywords:** titanium alloy, fracture toughness, stress corrosion cracking, corrosive environment

## Abstract

Ti-6Al-4V titanium alloy is extensively employed in deep-sea structural applications owing to its excellent corrosion resistance, while its extra-low-interstitial (ELI) variant provides higher fracture toughness and is commonly presumed to exhibit even better stress corrosion cracking (SCC) resistance. In this work, displacement-rate-dependent fracture toughness (K_Q_) measurements and failure analysis were performed for compact tension specimens machined from an engineering Ti-6Al-4V ELI plate with different orientations, tested in air and 3.5 wt.% NaCl solution over displacement rates of 0.0012–1.2 mm/min. In air, K_Q_ exhibits a pronounced loading-rate dependence, decreasing by more than 20% at low displacement rates relative to maximum rate, accompanied by quasi-cleavage features on the fracture surfaces indicative of hydrogen-assisted damage, likely arising from environmental or processing-related hydrogen uptake. In 3.5 wt.% NaCl solution, the minimum K_Q_ within the low-rate regime (0.0012–0.12 mm/min) is 58 MPa·m^0.5^, comparable to values reported for conventional Ti-6Al-4V under similar conditions. The pronounced rate dependence and transition toward cleavage-like fracture reveal a strong coupling between loading kinetics and environmental degradation. This work demonstrates that enhanced intrinsic toughness does not necessarily translate into superior SCC resistance and establishes loading rate as a critical factor governing the environmental fracture of Ti-6Al-4V ELI under marine conditions.

## 1. Introduction

Titanium alloys are widely used in load-bearing components across aviation, aerospace, and marine industries due to their high strength, low density, and excellent corrosion resistance [1]. Their corrosion resistance stems primarily from a dense, stable TiO_2_-rich passive film [2,3] that effectively blocks corrosive media, even in aggressive chloride-containing environments such as seawater. Consequently, commercial titanium alloys generally do not exhibit significant stress corrosion cracking (SCC) in seawater at room temperature. For instance, in slow strain-rate tests (SSRT) using smooth round bar specimens in a room-temperature salt solution, substantial elongation loss is rarely observed [4]. As a result, research on titanium alloy corrosion has largely focused on more extreme conditions, such as hot salt environments [5,6].

As a representative commercial titanium alloy, Ti-6Al-4V strikes an effective balance between mechanical performance and SCC resistance. Its typical α+β dual-phase structure provides high yield strength, fracture toughness, and fatigue resistance while avoiding the significant electrochemical heterogeneity associated with strong β-stabilizing elements. On this basis, to meet the stringent fracture toughness demands of critical load-bearing components, the extra-low interstitial (ELI) variant Ti-6Al-4V ELI has been developed, wherein reduced interstitial element contents (oxygen, nitrogen, carbon, and hydrogen) further enhance fracture toughness [7].

Although titanium alloys like Ti-6Al-4V ELI show little environmental sensitivity during SSRT on smooth specimens [8], they remain susceptible to environmentally assisted crack propagation and a lowered SCC threshold in seawater. This highlights a critical limitation: smooth-specimen data do not directly apply to actual structures with pronounced stress concentrations. In practice, SCC can initiate at features such as notches, particularly if protective coatings fail and allow seawater contact [9]. Moreover, the marine environment is characterized by complex composition, variable dissolved oxygen, and fluctuations in temperature and flow. Under such conditions, the loading rate plays a critical role in governing SCC behavior. At low displacement rates, sufficient time is available for hydrogen diffusion and crack tip electrochemistry, promoting local anodic dissolution, passive film destabilization, and micro-damage accumulation, all of which reduce crack propagation resistance. Therefore, whether Ti-6Al-4V ELI alloy exhibits superior SCC resistance across a range of loading rates in seawater environments requires further investigation.

This study systematically evaluates the fracture toughness of an engineering-grade Ti-6Al-4V ELI titanium alloy thick plate in air and 3.5 wt.% NaCl solution using compact tension (CT) specimens. The coupled influences of material orientation, displacement rate, and corrosive environment on fracture toughness and crack propagation are investigated to elucidate the underlying SCC mechanism under simulated marine conditions, thereby informing material selection and the service life assessment of critical load-bearing components.

## 2. Materials and Methods

### 2.1. Material and Specimen

**Figure 1:**
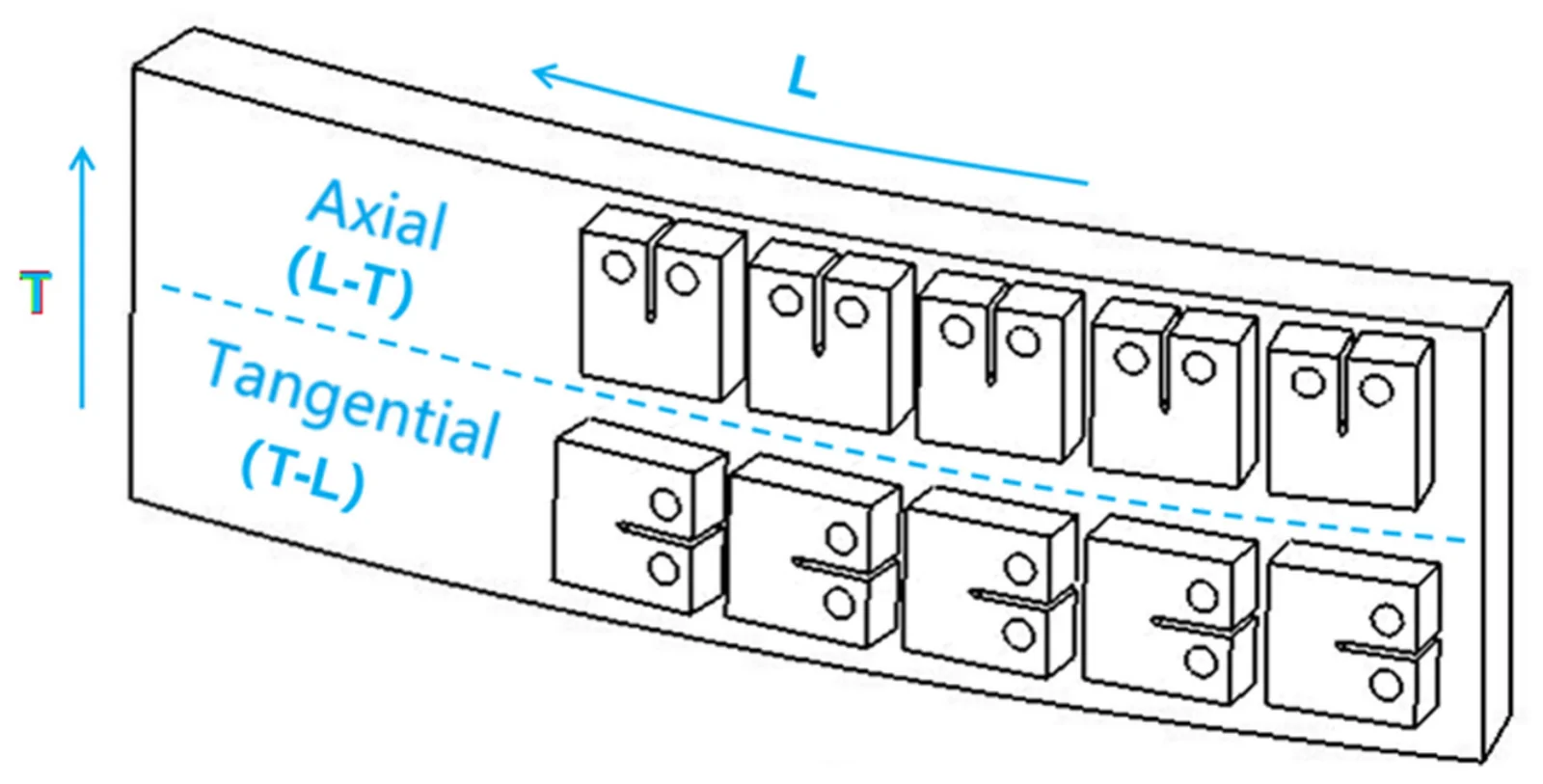
Illustration of the specimen orientation.

**Figure 2:**
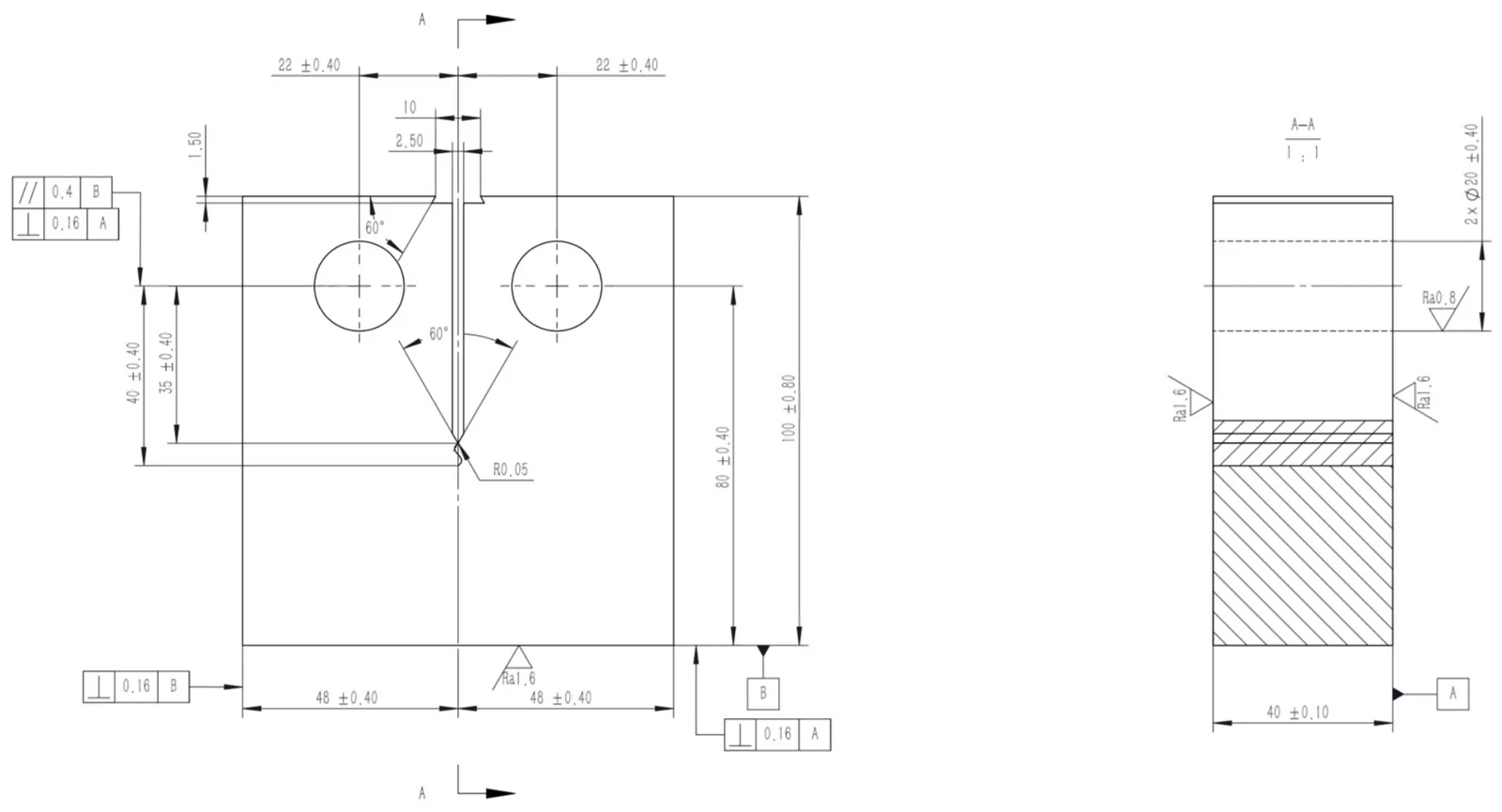
Geometry of the specimen (unit: mm).

The test material was taken from an arc-shaped Ti-6Al-4V ELI titanium alloy thick plate. The measured composition (in wt.%) was namely Al 6.51, V 4.36, Fe 0.22, O 0.12, N 0.0088, C 0.009, with Ti as the balance. The average grain size was measured to be ~20 μm [10]. The arc length direction is defined as the longitudinal direction (L), and the axial direction of the thick plate is defined as the transverse direction (T). Compact tension specimens were cut along two orientations: axial specimens corresponding to the L-T orientation and tangential specimens corresponding to the T-L orientation (Figure 1). The dimensions are shown in Figure 2.

### 2.2. Fracture Toughness Measurement

Fracture toughness tests in air and 3.5 wt.% NaCl solution were conducted using an Instron 8802 (Norwood, MA, USA) fatigue testing system. Crack opening displacement (COD) was recorded using an electronic extensometer, and specimen dimensions were measured with a digital caliper (±0.01 mm). Pre-cracking was performed in air at room temperature under sinusoidal cyclic loading (P_max_ = 18 kN, R = 0.05, f = 10 Hz) until the target crack length (a ≈ 40 mm) was achieved. Final specimen dimensions were obtained from multiple measurements at each location and averaged.

**Figure 3:**
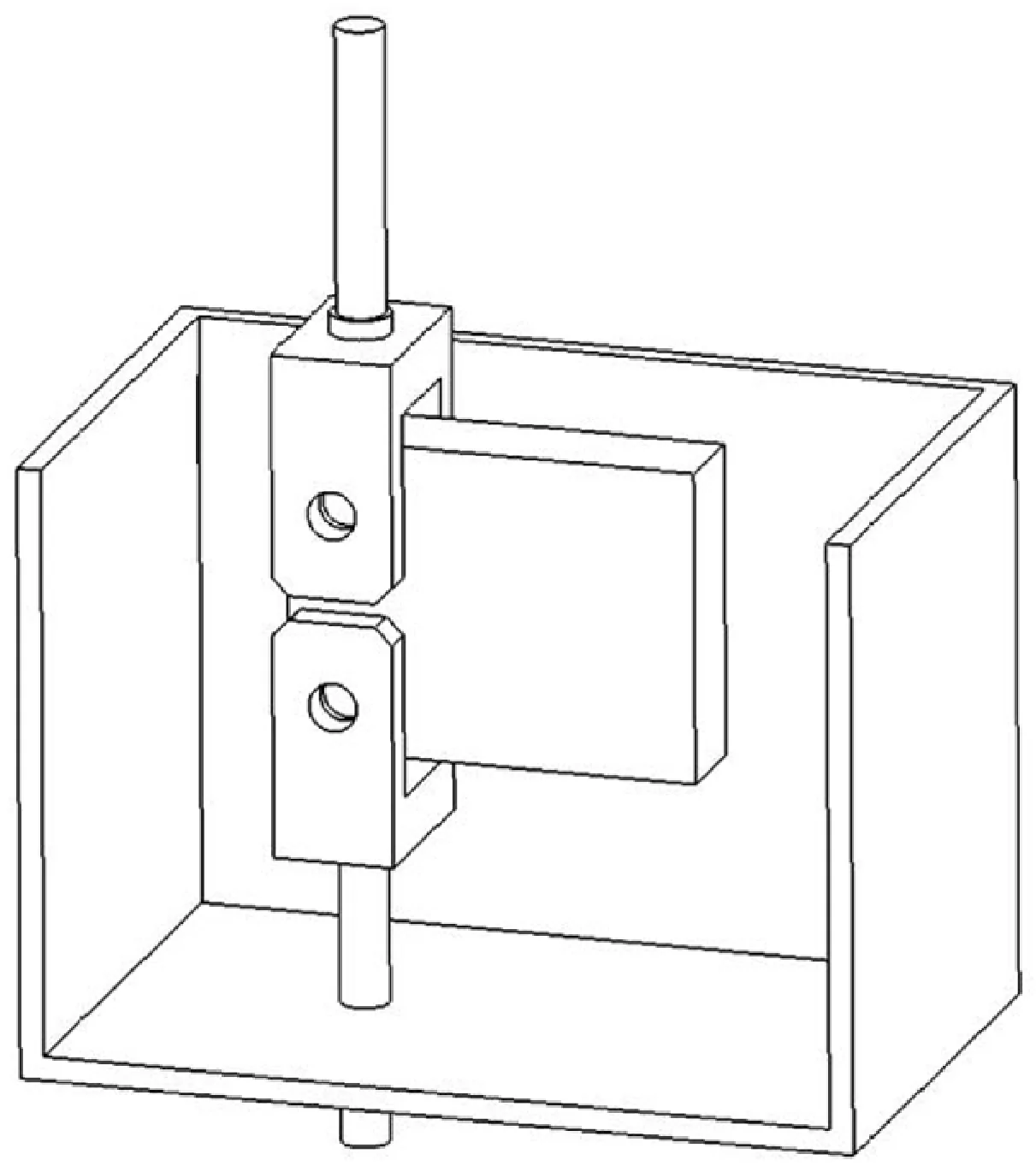
Schematic diagram of the experimental fixture and environmental chamber (front panel removed).

Fracture toughness tests were then conducted in both air and 3.5 wt.% NaCl solution using identical loading procedures and displacement rates. For environmental testing, CT specimens were mounted in a semi-enclosed corrosion cell allowing full immersion of the crack region and free fixture rotation (Figure 3); the extensometer was protected by a corrosion-resistant coating. The solution level was maintained above the crack tip throughout testing to ensure continuous exposure of the crack propagation zone to the corrosive medium.

The characteristic load, P_Q_, was determined from the experimental load–displacement (P–V) curves. The initial linear-elastic portion of each curve was fitted to obtain its slope, (P/V)_0_. A secant line with a slope equal to 95% of (P/V)_0_ was then constructed. The intersection of this secant line with the experimental curve was used to determine P_Q_, from which the provisional fracture toughness, K_Q_, was subsequently calculated.

The validity of K_Q_ as plane-strain fracture toughness, K_IC_, was assessed according to the criterion: B ≥ 2.5(K_IC_/*R*_p0.2_)^2^ where R_p0.2_ is the 0.2% proof stress. Since B = a = W/2 in the present study, the three requirements are equivalent. Assuming R_p0.2_ = 800 MPa and B = 40 mm, the maximum valid K_Q_ value was calculated to be 101 MPa·m^0.5^. Additional validity requirements, including K_max_/K_Q_ < = 1.1, were also satisfied. Fracture toughness characterizes the response of a cracked specimen under combined mechanical loading and corrosive attack, and its validity depends on maintaining sufficient plane-strain constraint. Because achieving full plane-strain conditions often requires large specimen dimensions, the constraint effect on the relationship between K_Q_ and K_IC_ is not considered herein. Nevertheless, it is known that SCC susceptibility generally decreases with reducing specimen size and constraint level [4]. All conditions were tested in triplicate.

### 2.3. Fracture Surface Observation

After fracture, the fracture morphologies were examined using an ultra-large-chamber scanning electron microscope (SEM, MIRA VP600, ELLCIE, Grevesmühlen, Germany). The system enables the direct observation of bulk specimens (up to 600 mm in diameter, 700 mm in height, and 300 kg in weight), thereby eliminating the need for sectioning and avoiding potential mechanical damage to the fracture surface while maintaining high-resolution imaging capability. Prior to observation, specimens were ultrasonically cleaned in a 75 vol.% ethanol–water solution for approximately 20 min to remove residual NaCl crystals and surface contaminants.

## 3. Results and Discussions

**Figure 4:**
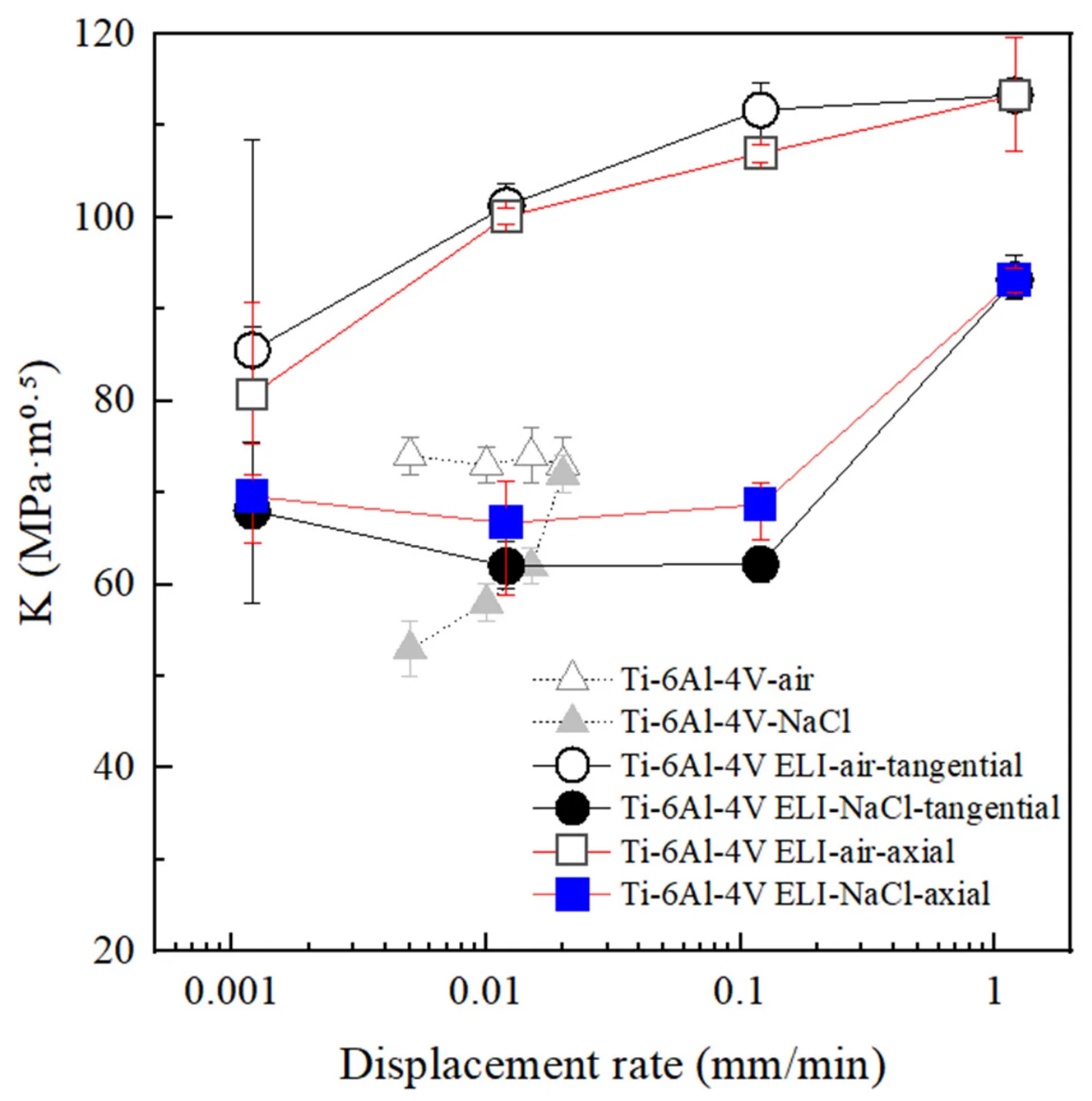
Fracture toughness under different displacement rates in air and NaCl solution. The triangle data points shown for the Ti-6Al-4V alloy are sourced from reference [8].

The fracture toughness (K_IC_/K_Q_) results of the Ti-6Al-4V ELI material in air and NaCl solution are shown in Figure 4, with comparative data for conventional Ti-6Al-4V alloy taken from the literature [8]. The corresponding values and validity assessments (with valid results denoted as K_IC_) are summarized in Table 1. As shown in Figure 4, in air, the fracture toughness exhibits a gradual decrease with decreasing displacement rate, accompanied by noticeable data scatter. In contrast, exposure to the NaCl solution leads to a pronounced reduction in fracture resistance across all loading rates. At low displacement rates (0.0012–0.12 mm/min), the minimum SCC-related fracture toughness of the Ti-6Al-4V ELI alloy is 58 MPa·m^0.5^, significantly lower than that measured in air. No discernible difference in fracture toughness is observed between tangential and axial orientations, indicating negligible anisotropy in fracture behavior under the present conditions. The weak orientation dependence of the measured fracture toughness in both air and corrosive media can be attributed, as discussed later, to the negligible role of pre-existing grain boundaries in crack propagation. Cracks propagate predominantly in a transgranular manner, which is less sensitive to local texture and grain size than intergranular cracking.

A relatively large scatter in K_Q_ was observed at the lowest displacement rate, particularly for the specimens tested in air. This scatter may arise from specimen-to-specimen variations in local microstructure and interstitial hydrogen distribution, which can affect hydrogen-assisted damage at low loading rates. Nevertheless, the average values at low displacement rates (0.0012–0.12 mm/min) fluctuate within 10% (Table 1), indicating a weak displacement-rate dependence. Using first-order scaling (100/37.5 ≈ 2.67), the present displacement rates are divided by 2.67 for comparison with the larger specimens; thus, 0.01 and 0.1 mm/min correspond to approximately 0.0037 and 0.037 mm/min, respectively. This scaling yields slightly higher K_Q_ values for the ELI grade than for conventional Ti-6Al-4V at comparable displacement rates.

It should be noted that the literature data shown in Figure 4 were obtained from smaller specimens (37.5 × 36 × 15 mm^3^). If crack tip strain rate is taken as the governing parameter, these results would correspond to higher equivalent strain rates for the larger specimens used in this work; thus, the literature data (triangular symbols) would effectively shift toward higher displacement rates. Owing to the complex relationship between crack tip strain rate and applied displacement rate, no quantitative conversion was performed in the present study.

Nevertheless, at relatively higher displacement rates (>0.01 mm/min), both datasets consistently indicate a strong dependence of fracture toughness on strain rate in NaCl solution. This behavior is generally attributed to the coupled effects of rate-dependent plastic deformation and hydrogen transport during SCC. In addition, the minimum K_Q_ measured in this study is slightly higher than that reported for Ti-6Al-4V alloy in the literature, suggesting that although interstitial reduction significantly improves air fracture toughness, its effect on SCC resistance remains limited.

**Figure 5:**
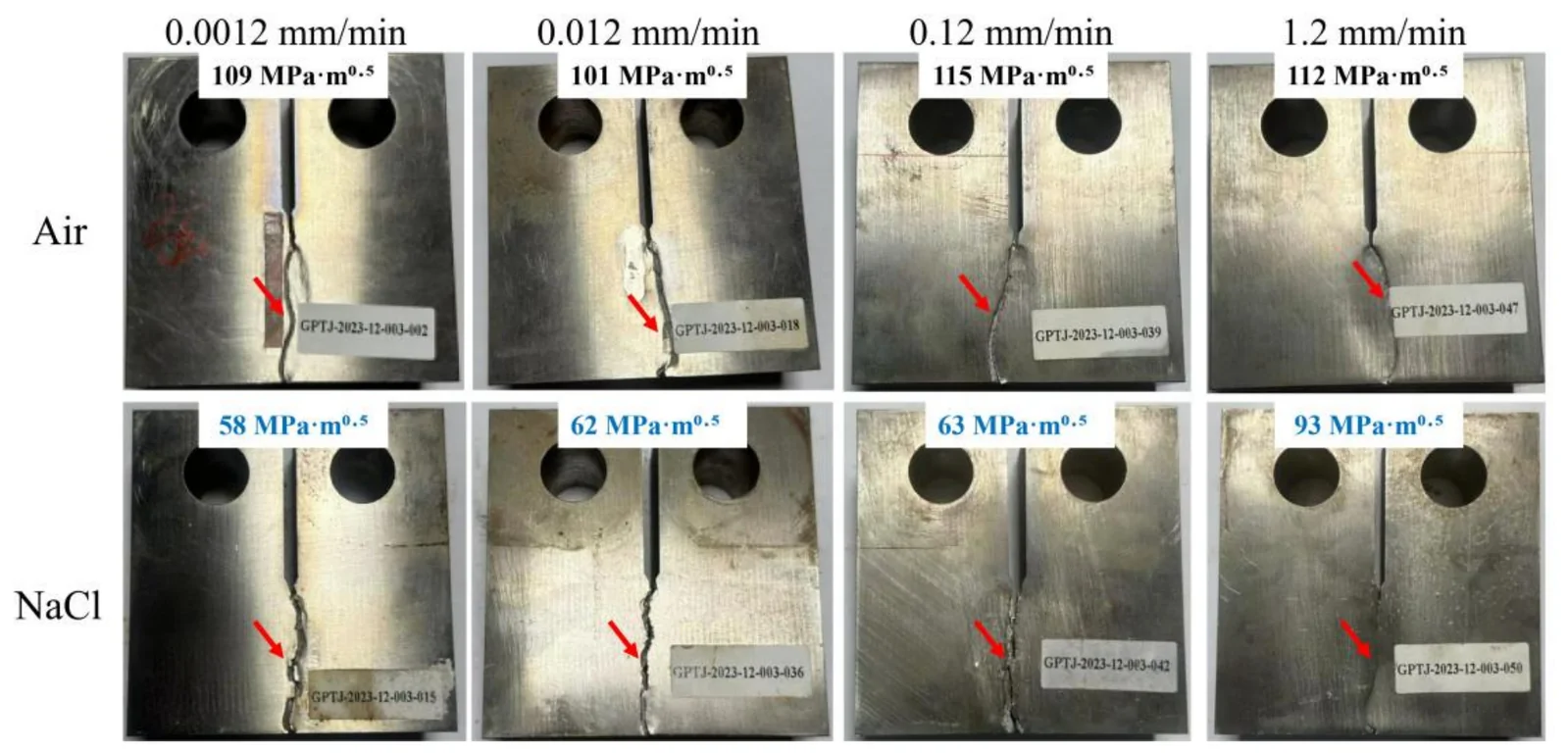
Photos of the tangential specimens after tests in air and NaCl solution.

**Figure 6:**
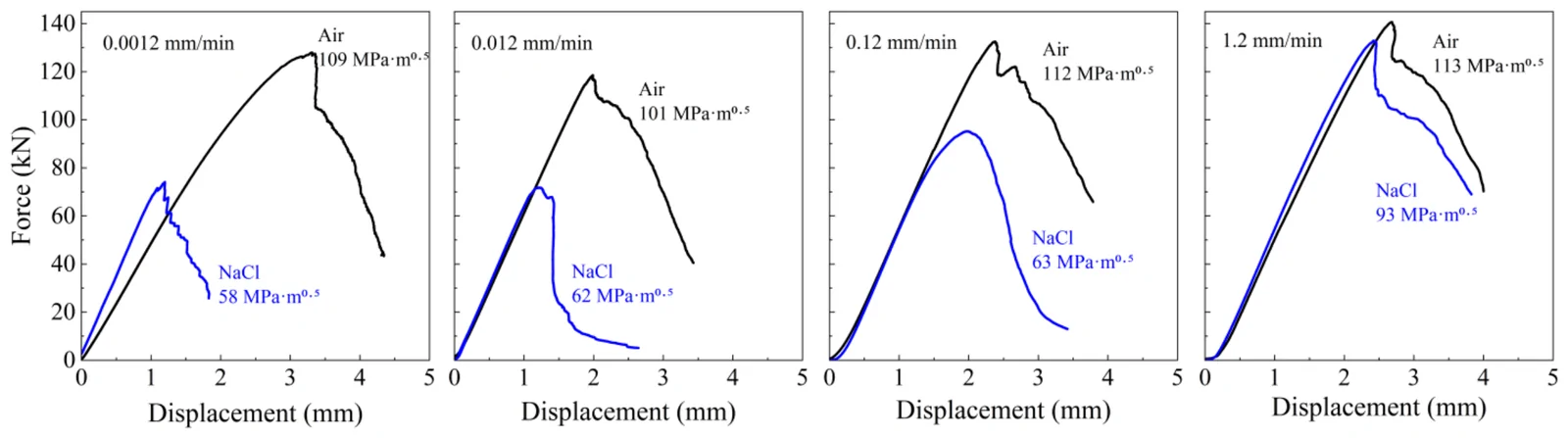
Force–displacement curves for the tangential specimens tested in air and NaCl solution at different displacement rates.

Figure 5 shows the macroscopic fracture morphologies of tangential specimens tested in 3.5 wt.% NaCl solution at different strain rates (arrows indicate the cracks). The SCC cracks propagate stably along the notch direction with negligible deflection; the crack plane deviates by less than ±10° from the initial notch plane. This confirms that crack growth remains predominantly mode I (opening mode), ensuring the validity of stress intensity factor evaluation based on linear-elastic fracture mechanics. Minor surface crack deflections observed in Figure 5 are associated with localized slant fracture, while the main crack path remains essentially aligned with the initial notch plane. Given the comparable mechanical response of tangential and axial specimens, subsequent analysis is focused on tangential specimens only. Figure 6 presents the load–displacement curves of tangential specimens tested at different displacement rates in air and NaCl solution. All curves exhibit a well-defined linear-elastic regime, confirming the applicability and reliability of the secant-based P_Q_ determination method, as described in the previous section.

Regarding the SCC mechanism of titanium alloys in chloride-containing environments, a growing body of evidence indicates that hydrogen-assisted damage plays a dominant role [11,12]. Under the synergistic action of tensile stress and aggressive chloride media, localized rupture of the passive film occurs, accompanied by intensified anodic dissolution and cathodic hydrogen evolution, thereby facilitating the generation, adsorption, and uptake of hydrogen at the crack tip. Driven by the steep stress gradients and triaxial stress fields ahead of the crack tip, a significant fraction of the absorbed hydrogen migrates and accumulates in highly stressed regions [13,14,15]. This localized hydrogen enrichment can weaken interatomic cohesion and reduce the critical energy barrier for crack advance, promoting premature crack initiation and unstable propagation. Concurrently, hydrogen may enhance localized plasticity degradation by impeding dislocation activity and promoting planar slip, thereby reducing the material’s capacity for crack tip plastic deformation [16,17]. As a result, crack growth is accelerated and the fracture mode progressively transitions toward cleavage-like characteristics [18,19,20].

**Figure 7:**
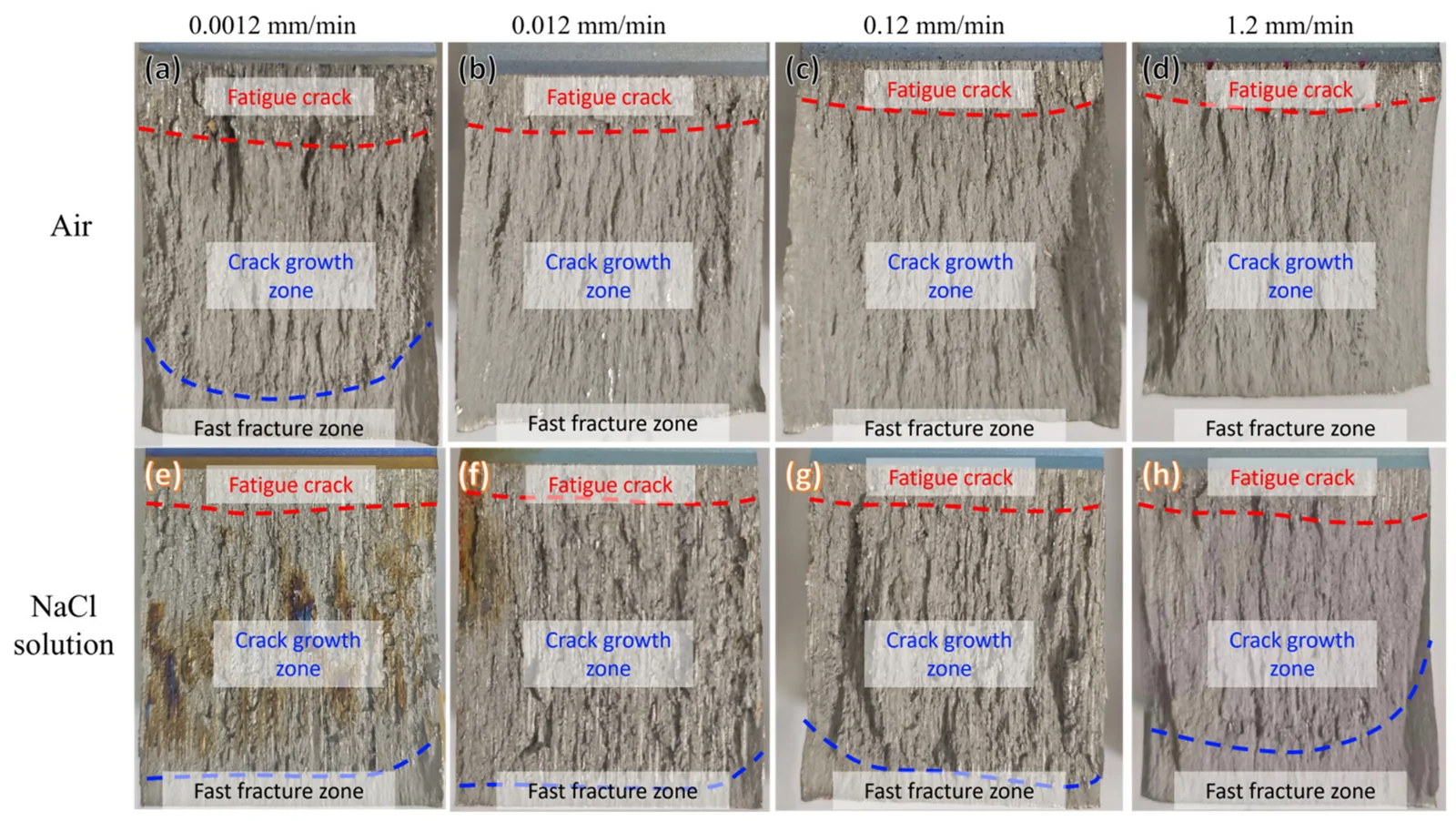
Photos of the tangential specimens after tests in NaCl solution. Dashed lines indicate the front of cracks. Air-tested specimens at displacement rates of 0.0012, 0.012, 0.12, and 1.2 mm/min are shown in (**a**–**d**), with the corresponding NaCl-solution results shown in (**e**–**h**), respectively.

Figure 7 shows the macroscopic fracture morphologies of tangential specimens tested in air and 3.5 wt.% NaCl solution at different displacement rates, with the pre-notch located at the top of each image. In air, a distinct macroscopic crack front is clearly observed at the lowest displacement rate (0.0012 mm/min), as indicated by the blue dashed line. At higher displacement rates (≥0.012 mm/min), micro-cracking becomes more dispersed and the transition between the macroscopic crack front and the dimple-dominated region is less well-defined. In contrast, specimens tested in NaCl solution exhibit a markedly different fracture response, with a well-defined SCC crack front observed at all displacement rates (blue dashed lines). Moreover, the locally bright regions visible on the fracture surfaces were identified by subsequent high-resolution SEM observations as cleavage-dominated fracture zones, indicative of brittle crack propagation under SCC conditions.

**Figure 8:**
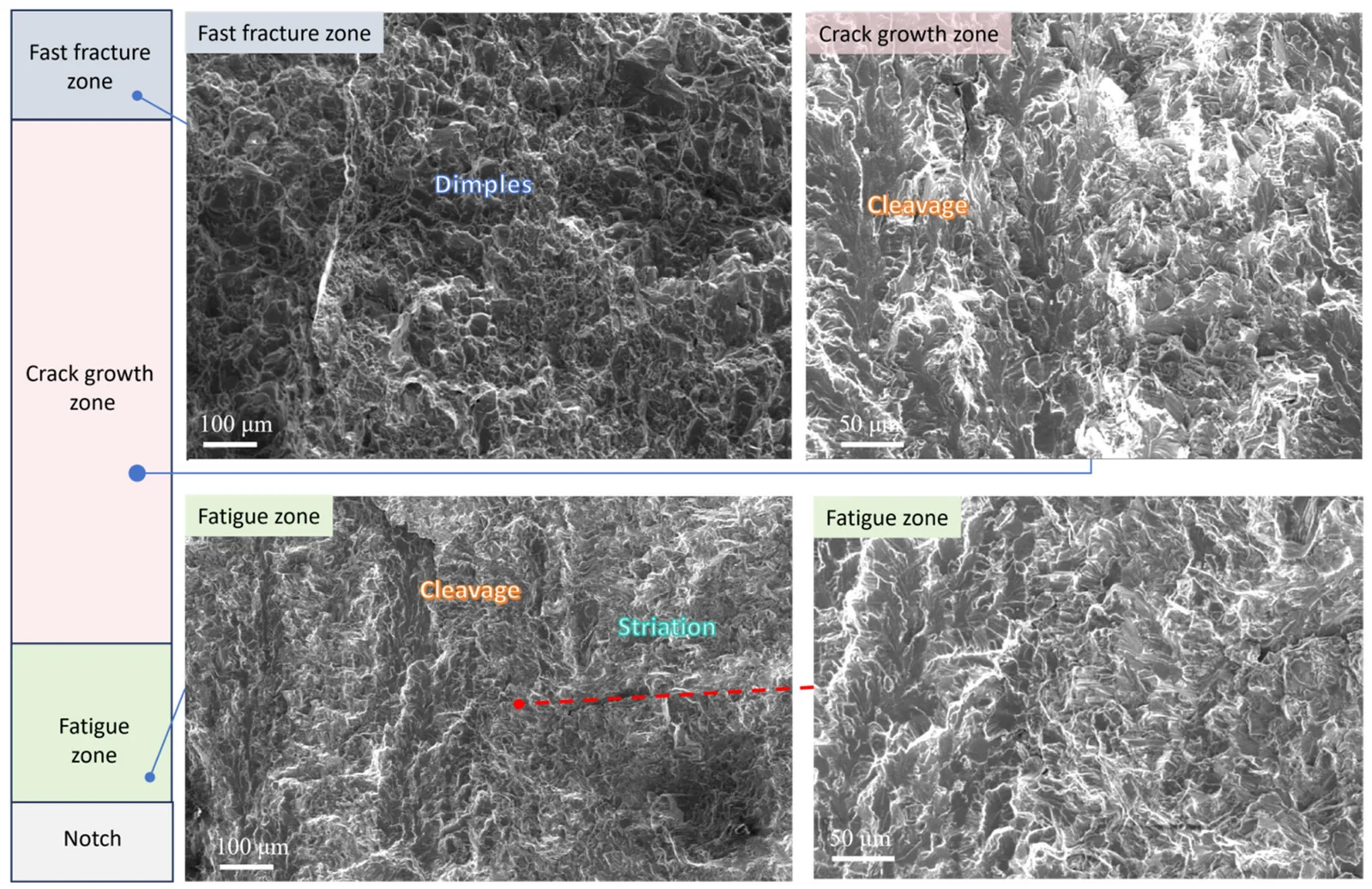
SEM fractographs of the tangential specimen tested in air (displacement rate: 0.012 mm/min). The dashed line indicates the location where the enlarged morphologies of the fatigue cracks were obtained.

**Figure 9:**
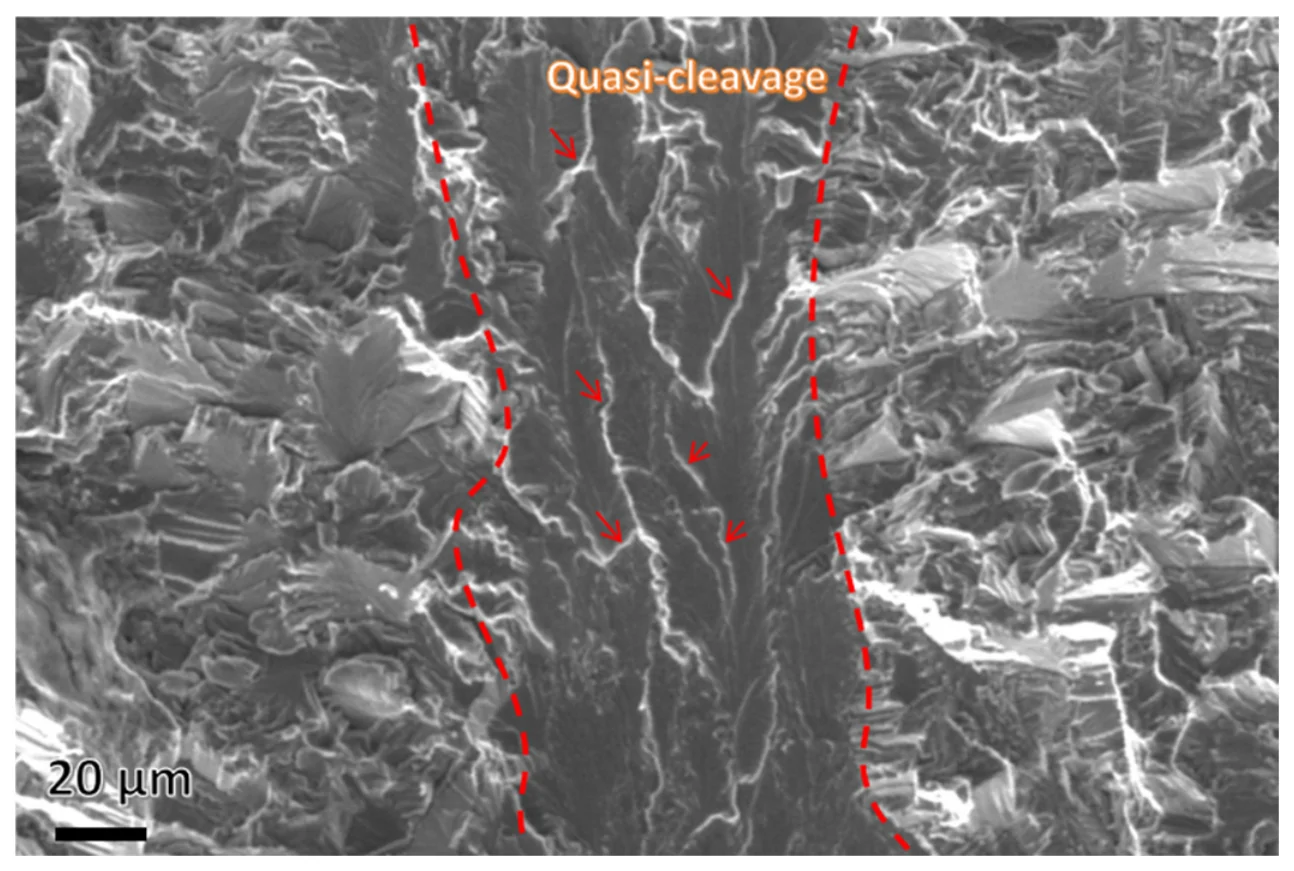
Representative SEM image showing the typical ridge-featured morphology of quasi-cleavage fracture in the fatigue crack region of the specimen tested in air at a displacement rate of 1.2 mm/min. Arrows point to the characteristic ridge features of quasi-cleavage cracks, while dashed lines indicate their boundaries.

**Figure 10:**
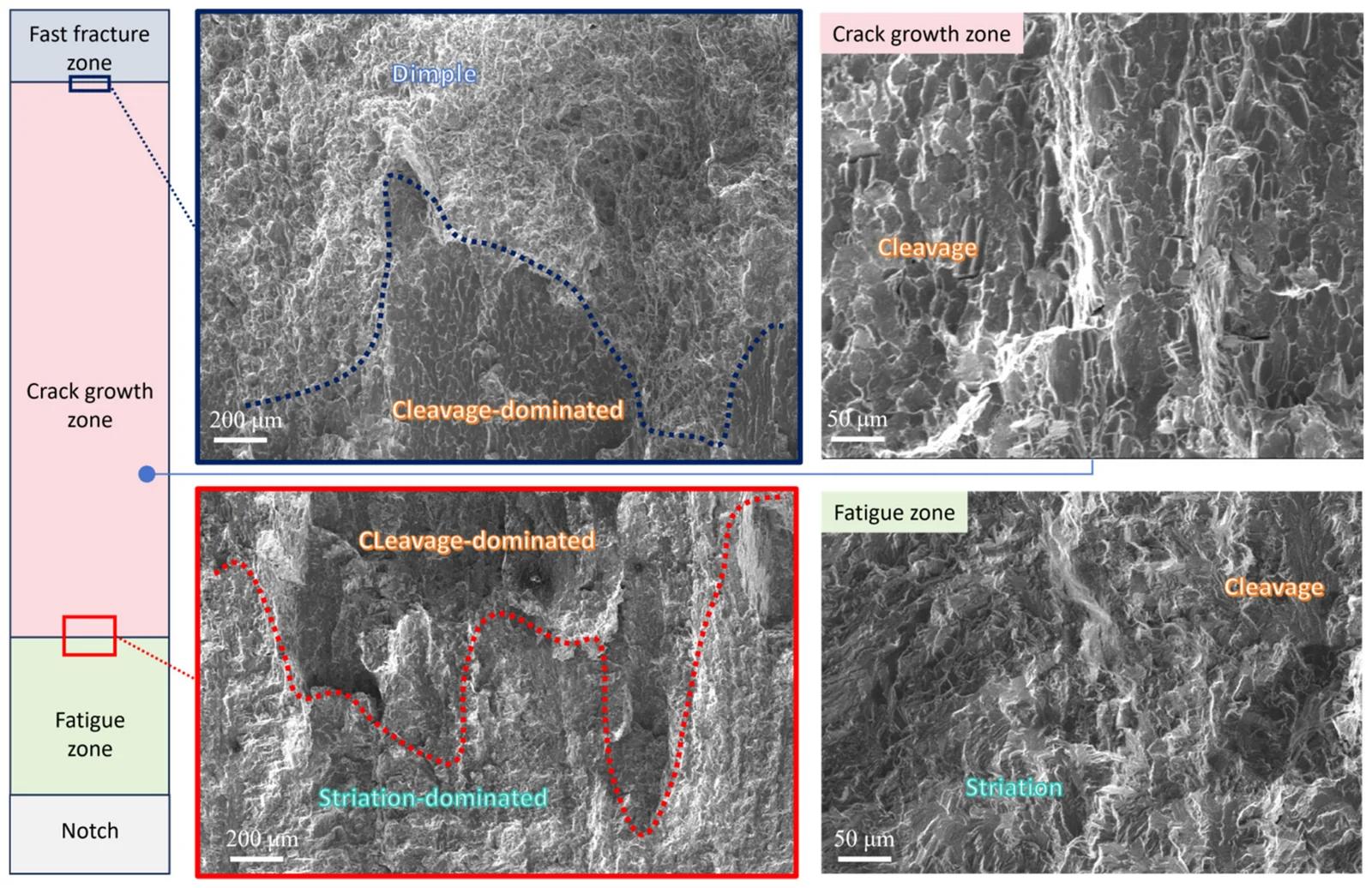
SEM fractographs of the tangential specimen tested in NaCl solution (displacement rate: 0.012 mm/min). Dashed lines indicate the front of cracks.

All fracture surfaces shown in Figure 7 were systematically examined, and representative SEM micrographs at a displacement rate of 0.012 mm/min are presented in Figure 8, Figure 9 and Figure 10. In air, crack propagation exhibits a typical ductile fracture mode, dominated by microvoid nucleation, growth, and coalescence, resulting in a surface covered by uniformly distributed dimples (Figure 8). The pre-fatigue crack region shows features of cleavage facets and fatigue striations. In contrast, specimens tested in NaCl solution display a progressive transition in fracture mode, from mixed fatigue striations and cleavage during fatigue crack growth to cleavage-dominated morphology under SCC conditions (Figure 10). Notably, these cleavage facets exhibit pronounced orientation dependence, typically forming elongated features with their long axis approximately parallel to the crack propagation direction, although macroscopically the fracture toughness is orientation-insensitive. Due to their planar shape, they produce strong reflection on the macroscopic fracture surface, corresponding to the bright elongated regions visible in Figure 7.

A high-magnification SEM image of the ridge features is shown in Figure 9. The closely spaced ridges indicate a quasi-cleavage fracture mode dominated by localized brittle-like crack propagation. Such morphology is consistent with transgranular crack growth and is often interpreted as being mediated by hydrogen-enhanced dislocation activity [21]. Interestingly, recent work has shown that such ridge features in titanium alloys are closely associated with highly localized plasticity mediated by nanoscale twinning [22]. The unexpected formation of quasi-cleavage cracks in both the fatigued zone and the crack propagation zone in air can be explained by hydrogen segregation. In room-temperature air, ductile fracture is generally expected under tensile loading, with failure dominated by microvoid nucleation, growth, and coalescence, producing a dimpled fracture surface, whereas the quasi-cleavage feature is considered strongly indicative of brittle fracture such as hydrogen embrittlement and stress corrosion cracking (a typical result of combined effects of internal and external hydrogen) [23]. It is noted that, for titanium alloys, existing studies have consistently reported the occurrence of cleavage or quasi-cleavage features during fatigue crack growth in air without the artificial introduction of internal or external hydrogen effects [24]. Nevertheless, the characterization of titanium hydrides remains challenging, and it has been suggested that hydrides in titanium alloys are nearly undetectable using conventional experimental techniques [25]. In addition, hydride precipitation tends to be highly localized, preferentially occurring at grain boundaries even at low average hydrogen contents, making it even more difficult to confirm the effect of hydrogen. As a result, most existing studies relied on artificial overcharging—up to thousands of mass ppm, which may far exceed the actual hydrogen content in engineering titanium plates—to induce observable hydrides. Furthermore, the effect of hydrogen content on the fracture morphologies of fatigued titanium specimens has also been shown to be complex, with no simple criterion for identifying the origin of features such as striations and cleavages. Although hydrides have been shown to induce a transition from typical fatigue-induced striations to cleavage features, it has also been confirmed that striations can still occur even at very high hydrogen concentrations where substantial hydride phase formation takes place, with hydrides confirmed beneath the striations using focused ion beam and transmission electron microscopy [26].

Despite the difficulty in directly confirming hydrogen’s role in the present study, the clear loading-rate dependency of fracture toughness in air is a strong indicator of hydrogen embrittlement, which is well known to be highly strain-rate-sensitive due to its strong dependence on hydrogen diffusion. At the crack tip, strong stress gradients promote hydrogen accumulation and may induce the formation of metastable hydrides in highly stressed regions [27,28]. Subsequent crack advance can fracture these brittle hydrides, while the newly created surfaces provide fresh sites for hydrogen re-accumulation and re-precipitation, forming a cyclic process of hydride formation, cracking, and reformation [29]. Because hydrogen transport is strongly strain-rate-dependent, the pronounced displacement-rate effect on K_Q_ observed in air in this study can be interpreted within this hydride-assisted cracking framework.

Hydrogen atoms preferentially segregate to phase, twin, and α/β interfaces, as well as to dislocations, where the local trapping sites can substantially increase the hydrogen concentration relative to the surrounding matrix. When the local hydrogen concentration exceeds the solubility limit, hydrogen can accumulate to a critical level for hydride formation or promote localized decohesion and slip localization along susceptible crystallographic planes. These highly concentrated regions can therefore act as preferential sites for crack nucleation and propagation. Moreover, the associated stress and chemical-potential gradients provide short-circuit pathways for subsequent hydrogen redistribution, accelerating hydrogen transport toward the advancing crack tip and establishing a positive feedback between hydrogen accumulation and crack growth.

To provide a quantitative evaluation of the possibility of internal hydrogen uptake, the relationship between hydride thickness and the average hydrogen concentration in α+β titanium alloys can be established as follows. It is assumed that, in engineering titanium plates after long-term air storage where diffusible hydrogen is considered negligible, most retained hydrogen is contained within hydrides that precipitate preferentially along α/α grain boundaries and α/β phase interfaces, forming thin interfacial layers with a uniform thickness, t. The hydride phase is assumed to be δ-TiH_1.5_, corresponding to a hydrogen concentration of approximately 30,600 mass ppm. The residual hydrogen concentration in the matrix is denoted as C_m_. For an equiaxed α microstructure with an average grain size d_α_, the grain-boundary area density can be approximated as S_gb_ = 2/d_α_. To account for hydride precipitation at α/β interfaces, an interface factor k_ab_ is introduced, representing the ratio of α/β interfacial area to α grain-boundary area. The total interfacial area density is therefore given by S_tot_ = S_gb_(1 + k_ab_). In addition, a dispersion factor f_d_ is defined to describe the fraction of interfaces covered by hydrides. A value of f_d_ = 1 corresponds to continuous hydride coverage along all eligible interfaces, whereas f_d_ = 0.5 indicates that only 50% of the available interfacial area is occupied by hydrides. The effective interfacial area density is thus S_eff_ = f_d_ · S_tot_. Assuming that the hydride layer thickness is much smaller than the characteristic microstructural dimensions, the hydride volume fraction can be approximated as f_h_ = S_eff_ · t. The average hydrogen concentration of the alloy is then obtained as C_avg_ = C_m_(1 − f_h_) + C_Hhyd_ · f_h_, where C_Hhyd_ is the hydrogen concentration within the hydride phase. Substituting f_h_ = S_eff_ · t yields C_avg_ = C_m_ + (C_Hhyd_ − C_m_)S_eff_ · t, indicating that the average hydrogen concentration increases approximately linearly with hydride thickness. For a representative Ti-6Al-4V microstructure with an α grain size of 20 μm, *k*_ab_ = 1, and continuous hydride coverage (f_d_ = 1), the model predicts that hydride layers with thicknesses of approximately 15 nm and 80 nm correspond to average hydrogen concentrations of 100 ppm and 500 ppm, respectively. Conversely, if only 20% of the interfaces are covered by hydrides (f_d_ = 0.2), the required hydride thickness increases by a factor of five. These calculations suggest that, although locally high hydrogen atom concentrations exceeding hydrogen solubility is required for hydride precipitation, extensive hydride films distributed along grain boundaries and α/β interfaces can precipitate (possibly with tens of nms) for average hydrogen concentrations commonly measured in engineering-grade Ti-6Al-4V alloys.

In terms of deformation mechanism and its interaction with stress corrosion cracking, twinning plays a critical role in the plastic deformation of titanium alloys and can promote the formation of dynamically recrystallized nanograins in highly strained regions such as crack tips [30,31]. The resulting nanoscale microstructures and their crystallographic orientation distributions exert complex multiscale effects on SCC behavior. Although crack initiation in titanium alloys is often reported to occur preferentially on basal and prismatic planes, crack propagation paths are considerably more complex and strongly influenced by deformation twins. Since twin boundary formation is primarily governed by the local stress state and crystallographic orientation, it is only weakly dependent on the initial interstitial content. Accordingly, even in low-interstitial Ti-6Al-4V ELI alloys designed to improve intrinsic fracture toughness and resistance to fatigue crack initiation, dense twin boundary networks can still develop at crack tips under corrosive conditions. These interfaces may interact with hydrogen generated by corrosion processes, potentially serving as preferential sites for hydrogen segregation or hydride formation. This provides a mechanistic rationale for the observation that Ti-6Al-4V ELI does not exhibit a substantially improved SCC resistance compared to conventional Ti-6Al-4V in corrosive environments.

From an electrochemical perspective, the solution confined within the pre-crack exhibits typical crevice corrosion characteristics. Restricted oxygen diffusion establishes a differential aeration cell. The resulting autocatalytic process leads to progressive acidification within the crack, with pH values potentially decreasing to two or lower, which destabilizes the passive film and triggers severe crevice corrosion [32]. Concurrently, the high triaxial stress field at the crack tip enhances anodic dissolution, promotes hydrogen evolution, and facilitates hydrogen uptake, thereby coupling electrochemical degradation with mechanical driving forces. While anodic dissolution continuously damages the passive film, cathodically generated hydrogen serves as the primary contributor to brittle crack propagation. These synergistic interactions constitute the fundamental mechanism governing SCC initiation and growth, and also explain the contrasting behavior of titanium alloys, which show good resistance in smooth-specimen SSRT tests but pronounced susceptibility in pre-cracked conditions. From a hydrogen embrittlement standpoint, hexagonal close-packed titanium alloys may exhibit hydrogen-enhanced plasticity at low hydrogen concentrations [33], whereas embrittlement dominates above a critical hydrogen level. It is therefore inferred that hydrogen accumulation at the crack tip under corrosive conditions exceeds this critical threshold, thereby accelerating brittle fracture.

Despite the important role of corrosion reactions in accelerating crack propagation, corrosion-film analysis remains challenging because the native passive film on Ti-6Al-4V ELI is extremely thin and can be readily altered during specimen preparation and analysis. Conventional techniques such as X-ray photoelectron spectroscopy and transmission electron microscopy may therefore yield ambiguous results, as ion milling, vacuum exposure, and electron-beam irradiation can modify or damage the film. Moreover, an oxide/hydroxide layer inevitably forms upon aqueous exposure, making it difficult to distinguish the native passive film from preparation- or exposure-induced surface products. Accordingly, such analyses should be interpreted with caution when assessing the role of corrosion films in stress corrosion cracking.

In terms of hydride precipitation induced by possible environment- or processing-related hydrogen contamination, it is unsurprising that local hydrogen segregation promotes preferential hydride formation along grain boundaries and phase boundaries, which can occur even when the bulk hydrogen concentration remains well below the engineering specification for chemical composition control (e.g., 125 mass ppm for Ti–6Al–4V ELI) or the hydrogen solubility limit in α-Ti (>200 mass ppm), thereby easily evading routine industrial detection. In engineering titanium plates subjected to long-term air storage, diffusible hydrogen is arguably negligible, and the majority of hydrogen exists in an irreversible form (i.e., hydride phases).

## 4. Conclusions

Based on the systematic investigation of an engineering Ti-6Al-4V ELI titanium alloy plate in air and 3.5 wt.% NaCl solution, the main conclusions are summarized as follows:

1. Ti-6Al-4V ELI exhibits evident SCC susceptibility in NaCl solution, which is strongly dependent on displacement rate. In the low-rate regime (0.0012–0.12 mm/min), the minimum average KQ is 62 ± 2 MPa·m^0.5^, significantly lower than that in air (81–113 MPa·m0.5) and comparable to reported values for conventional Ti-6Al-4V alloy.
2. Notably, KIC/KQ also decreases markedly with decreasing displacement rate in air, accompanied by a transition toward quasi-cleavage fracture. This low-rate embrittlement in the ELI grade highlights a previously underappreciated susceptibility to hydrogen-assisted damage even under nominally inert conditions. The estimation of irreversible hydrogen segregation indicates the likely formation of thin hydrides with a low overall hydrogen concentration below the engineering threshold for the Ti-6Al-4V ELI alloy.
3. Interstitial reduction enhances intrinsic toughness but provides limited improvement in SCC resistance, indicating that crack propagation from pre-existing defects dominates service failure risk.

## Figures and Tables

**Table 1:** K_Q_ or K_IC_ values of Ti-6Al-4V ELI alloy tested in various environments and at different displacement rates.

Environment	Displacement Rate, mm/min	K_max_, MPa·m^0.5^	K_Q_, MPa·m^0.5^	K_Q_ or K_IC_	Average K_Q_, MPa·m^0.5^
Axial					
Air	0.0012	98	75	K_Q_	81
		104	91		
		99	76		
	0.012	102	100	K_IC_	100
		102	101		
		103	99		
	0.12	109	106	K_Q_	100
		108	86		
		111	108		
	1.2	113	113	K_Q_	113
		122	120		
		107	107		
3.5 wt.% NaCl solution	0.0012	90	87	K_IC_	74
		91	67	K_Q_	
		78	68		
		78	74	K_IC_	
	0.012	77	75	K_IC_	67
		72	62	K_Q_	
		77	63		
	0.12	95	66	K_Q_	69
		92	67		
		88	72		
	1.2	115	94	K_Q_	93
		116	92		
		115	93		
Tangential					
Air	0.0012	114	109	K_Q_	85
		96	79		
		92	79		
		92	75		
	0.012	106	104	K_Q_	101
		104	101	K_IC_	
		101	99		
	0.12	116	115	K_Q_	112
		114	112		
		108	108		
	1.2	115	114	K_Q_	113
		118	113		
		115	112		
3.5 wt.% NaCl solution	0.0012	92	88	K_IC_	68
		64	58		
		66	58	K_Q_	
	0.012	62	62	K_IC_	62
		66	65		
		65	60		
	0.12	81	63	K_Q_	62
		85	64		
		81	60		
	1.2	117	91	K_Q_	93
		119	96		
		114	93		

## Data Availability

The original contributions presented in this study are included in the article. Further inquiries can be directed to the corresponding author.

## References

[1] Veiga C., Davim J.P., Loureiro A. (2012). Properties and applications of titanium alloys: A brief review. Rev. Adv. Mater. Sci..

[2] Pouilleau J., Devilliers D., Garrido F., Durand-Vidal S., Mahé E. (1997). Structure and composition of passive titanium oxide films. Mater. Sci. Eng. B.

[3] Simka W., Sadkowski A., Warczak M., Iwaniak A., Dercz G., Michalska J., Maciej A. (2011). Characterization of passive films formed on titanium during anodic oxidation. Electrochim. Acta.

[4] Blackburn M., Feeney J., Beck T. (1973). Stress-corrosion cracking of titanium alloys. Advances in Corrosion Science and Technology.

[5] Shi Y., Joseph S., Saunders E.A., Sandala R.S., Walker A., Lindley T.C., Dye D. (2021). AgCl-induced hot salt stress corrosion cracking in a titanium alloy. Corros. Sci..

[6] Sinigaglia D., Taccani G., Vicentini B., Dallaspezia G. (1978). Electrochemical behavior of titanium and some titanium alloys under tensile stress in boiling sulfuric acid and acidic chloride solutions. J. Electrochem. Soc..

[7] Ogden H., Jaffee R. (1955). The Effects of Carbon, Oxygen, and Nitrogen on the Mechanical Properties of Titanium and Titanium Alloys.

[8] Barella S., Mapelli C., Venturini R. (2005). Investigation about the stress corrosion cracking of Ti-6Al-4V. Metall. Sci. Tecnol..

[9] Hidalgo Viteri J., Cotolan N., Lupan A., Brânzanic A.M.V., Turdean G.L. (2024). A poly(methyl methacrylate)–ibuprofen composite film as anticorrosive coating of Ti–6Al–4 V surface. J. Solid State Electrochem..

[10] Zhang B., Liu J., Rui S.-S., Chen W., Sun C. (2026). Fatigue properties and influential factors of Ti-6Al-4V ELI titanium alloy used in deep-sea pressure hull structure. Eng. Fract. Mech..

[11] Powell D., Scully J. (1968). Stress corrosion cracking of alpha titanium alloys at room temperature. Corrosion.

[12] Hollis A., Scully J. (1993). The stress corrosion cracking and hydrogen embrittlement of titanium in methanol-hydrochloric acid solutions. Corros. Sci..

[13] Sofronis P., McMeeking R.M. (1989). Numerical analysis of hydrogen transport near a blunting crack tip. J. Mech. Phys. Solids.

[14] Wang Y., Sharma B., Xu Y., Shimizu K., Fujihara H., Hirayama K., Takeuchi A., Uesugi M., Cheng G., Toda H. (2022). Switching nanoprecipitates to resist hydrogen embrittlement in high-strength aluminum alloys. Nat. Commun..

[15] Wang Y., Tang J., Fujihara H., Adachi N., Todaka Y., Xu Y., Saha M., Sasaki T., Shimizu K., Hirayama K. (2024). Advancing the hydrogen tolerance of ultrastrong aluminum alloys via nanoprecipitate modification. Corros. Sci..

[16] Hu S., Tian Z., Wang Y., Hu H., Li X., Liu Q., Liu D., Li Y., Cheng G. (2023). Combined impact of elastic stress, prestrain and electrochemical charging on the hydrogen-induced cracking of high-strength steel. Int. J. Hydrogen Energy.

[17] Kim J., Plancher E., Tasan C.C. (2020). Hydrogenation-induced lattice expansion and its effects on hydrogen diffusion and damage in Ti–6Al–4V. Acta Mater..

[18] Kim J., Hall D., Yan H., Shi Y., Joseph S., Fearn S., Chater R.J., Dye D., Tasan C.C. (2021). Roughening improves hydrogen embrittlement resistance of Ti-6Al-4V. Acta Mater..

[19] Wang Y., Toda H., Fujihara H., Shimizu K., Hirayama K., Takeuchi A., Uesugi M. (2025). Role of retrogression and reaging in suppressing hydrogen-induced transgranular cracking in 7xxx Al alloys. Scr. Mater..

[20] Martin M.L., Fenske J.A., Liu G.S., Sofronis P., Robertson I.M. (2011). On the formation and nature of quasi-cleavage fracture surfaces in hydrogen embrittled steels. Acta Mater..

[21] Robertson I.M., Sofronis P., Nagao A., Martin M.L., Wang S., Gross D.W., Nygren K.E. (2015). Hydrogen Embrittlement Understood. Metall. Mater. Trans. B.

[22] Wang Y., Liu Q., Shao Y., Zhao Q., Su H., Wang J., Lin Y., Liu Z., Sun C. (2026). Mechanisms of sustained load cracking in high vacuum environment and stress corrosion cracking for Ti-6Al-4V ELI alloy in deep-sea structures. Corros. Sci..

[23] Tang J., Wang Y., Fujihara H., Shimizu K., Hirayama K., Ebihara K., Takeuchi A., Uesugi M., Toda H. (2024). Stress corrosion cracking induced by the combination of external and internal hydrogen in Al-Zn-Mg-Cu alloy. Scr. Mater..

[24] Meyn D., Brooks E. (1981). Stress-Corrosion Cracking in Titanium Alloys. Fractography Mater. Sci..

[25] Bache M.R. (2003). A review of dwell sensitive fatigue in titanium alloys: The role of microstructure, texture and operating conditions. Int. J. Fatigue.

[26] Moreira L.C.M. (2024). Hydrogen Embrittlement of Titanium Alloys: Impact of Hydrogen on the Mechanical Behavior Under Low Cycle Fatigue. Ph.D. Thesis.

[27] Lufrano J., Sofronis P., Birnbaum H.K. (1996). Modeling of hydrogen transport and elastically accommodated hydride formation near a crack tip. J. Mech. Phys. Solids.

[28] von Pezold J., Lymperakis L., Neugebeauer J. (2011). Hydrogen-enhanced local plasticity at dilute bulk H concentrations: The role of H–H interactions and the formation of local hydrides. Acta Mater..

[29] Shishvan S.S., Csányi G., Deshpande V.S. (2020). Hydrogen induced fast-fracture. J. Mech. Phys. Solids.

[30] Suresh S. (1998). Fatigue of Materials.

[31] Sun C., Wu H., Chi W., Wang W., Zhang G.-P. (2023). Nanograin formation and cracking mechanism in Ti alloys under very high cycle fatigue loading. Int. J. Fatigue.

[32] Watson M., Postlethwaite J. (1990). Numerical simulation of crevice corrosion of stainless steels and nickel alloys in chloride solutions. Corrosion.

[33] Froes F., Senkov O., Qazi J. (2004). Hydrogen as a temporary alloying element in titanium alloys: Thermohydrogen processing. Int. Mater. Rev..

